# Chemical constituents, antioxidant and cytotoxicity properties of *Leonotisleonurus* used in the folklore management of neurological disorders in the Eastern Cape, South Africa

**DOI:** 10.1007/s13205-020-2126-5

**Published:** 2020-02-27

**Authors:** Sipho Tonisi, Kunle Okaiyeto, Heinrich Hoppe, Leonard V. Mabinya, Uchechukwu U. Nwodo, Anthony I. Okoh

**Affiliations:** 1 SAMRC Microbial Water Quality Monitoring Centre, University of Fort Hare, Alice, 5700 South Africa; 2 Applied and Environmental Microbiology Research Group (AEMREG), Department of Biochemistry and Microbiology, University of Fort Hare, Alice, 5700 South Africa; 3 Department of Biochemistry and Microbiology, Rhodes University, Grahams Town, 6140 South Africa

**Keywords:** *Leonotisleonurus*, Chemical compositions, Antioxidant, Cytotoxicity properties

## Abstract

In the present study, we evaluated the phytochemical compounds and antioxidant properties of chloroform, ethanol and acetone extracts for leaves and flowers of *Leonutus* *leonurus* (*L.* *leonurus*) alongside with their cytotoxic effects on human cervical carcinoma (HeLa) cell lines. The phytochemical compounds present in the leaves and flowers of *L.* *leonurus* included; phenolics, flavonoids and alkaloids. Their radicals scavenging effects against 2, 2-diphenyl-1-picrylhydrazyl [DPPH] 2,2′-azinobis-(3-ethylbenzothiazoline-6-sulphonate) [ABTS·^+^], hydrogen peroxide, nitric oxide as well as metal chelating activities showed dose-dependent activities. Gas chromatography-mass spectrometry (GCMS) analyses revealed the presence of important bioactive compounds, which are associated with antioxidant; and the extracts exhibited toxicity effect against HeLa cells. The findings from this study divulge extracts of *L.* *leonurus* as prospective sources of antioxidant and anticancer agents; and hence, further study on their neuroprotective potentials becomes imperative.

## Introduction

Medicinal plants have always been regarded as valuable sources of potential drugs that could be used against a number of dreadful diseases in human beings (Newman and Cragg 2012). The increasing interest in medicinal plants stems from the vital role they play in the health of indigenous people in a traditional setting. It is commonly stated that the medicinal value of a plant entirely depends on the presence of organic compounds, also referred to as phytochemicals (Dhandapani and Sabna 2008). Phytochemicals are referred to as secondary metabolites produced by plants during growth and development. These phytochemicals are responsible for a plant’s aroma, color and flavor as well as protection during infection and environmental stress (Kumar 2019). Such phytochemicals include; phenolics, flavonoids, alkaloids, steroids, terpenoids, saponins, tannins, anthraquinones and glycosides (Dhandapani and Sabna 2008). The therapeutic properties medicinal plants possess have led to the exploration of secondary metabolites as alternative drugs with less side effects than synthetic ones. Besides other medicinal properties such as anti-inflammatory, anti-bacterial, anti-diabetic activity, one prominent feature that attracts researchers to natural bioactive compounds is their antioxidant potential that enables them to scavenge the production of free radicals, which are molecules associated with many life-threatening diseases (Sati et al. 2010).

Free radicals are described as highly reactive molecules with an unpaired electron in their valence (Senguttuvan et al. 2014). The production of free radicals occurs during normal metabolism; however, excessive amounts can cause cellular and tissue damage through a process called oxidative stress (Sen et al. 2010). Oxidative stress can lead to a variety of degenerative diseases such as inflammation, heart diseases, cancer, lung damage and neurodegenerative disorders (Cavalcanti et al. 2006). Therefore, for a bioactive compound to be referred to as an antioxidant, it must be able to delay or inhibit oxidative damage by scavenging free radicals (Yamagishi and Matsui 2011). For the past decades, medicinal plants emerged as the richest sources of natural antioxidants due to their role in the treatment of human diseases (Upadhyay et al. 2010). Another interesting property of natural antioxidants is their low toxicity, thus making them safer to use than synthetic drugs (Meenakshi et al. 2011). In essence, if a plant possesses good antioxidant activity it stands a good possibility of effectively treating a variety of human diseases where oxidative stress is highly implicated.

*Leonotis* *leonurus,* commonly known as the “wild dagga” in the Eastern Cape Province of South Africa, is a plant species belonging to the family of Lamiaceae and is largely distributed in the Southern Africa region (Nsuala et al. 2015). In folkloric medicine, this plant has been documented to have health benefits for indigenous people around the Eastern Cape Province, South Africa (Scott et al. 2004; Mazimba 2015). The traditional use of *L.* *leonurus* includes, amongst others, as therapy for pain management caused by snakebite, headache, wounds, bronchitis, high blood pressure, common cold, influenza, chest pains, epilepsy, menstrual cycle period pains and constipation (McGaw et al. 2000). The leaves of the plant are often used externally as a treatment for itchy skin and eczema. The stem is commonly used to prepare an aqueous extract that is ingested for cleansing the blood of any impurities (Watt and Breyer-Brandwijk 1962). Infusions made from seeds and flowers, leaves or stems are regularly used as tonics for tuberculosis, high blood pressure, jaundice, muscular cramps, diabetes, diarrhoea, viral hepatitis and dysentery (Nsuala et al. 2015). Also, *L.* *leonurus* has been reported to demonstrate anti-inflammatory, antioxidant, anti-diabetic and hepatoprotective properties (El-Ansari et al. 2009; Jimoh et al. 2010; Mazimba, 2015).

Previous studies have revealed that the antioxidant potential of *L.* *leonurus* could be related to the presence of phenolics, alkaloids and flavonoids, which are bioactive compounds associated with anti-cancerous, anti-inflammatory and wound healing properties (Ojewole 2005; Jimoh et al. 2010; Popoola et al. 2013). However, the full antioxidant potential of this plant remains underexplored as there are few studies reported. It is, therefore, plausible that the chemical composition of *L.* *leonurus* might possess bioactive compounds that may play a role in scavenging highly reactive molecules that tend to initiate as well as exacerbate the pathology of many health conditions, especially neurological disorders. It is, therefore, a matter of necessity to explore this phenomenon, as it would expound on the existing scientific knowledge about this plant. In the present study, the phytochemical compounds and antioxidant properties of extracts of the leaf and flower parts of *L.* *leonurus* were investigated and thereafter, their cytotoxicity potential against HeLa cells was assessed.

## Materials and methods

### Plant collection and authentication

The ethical clearance certificate that permits the collection of the plants was obtained from the University of Fort Hare with the certificate reference number MAB021STON01. The plants used for this study were harvested from Hogsback in Raymond Mhlaba Local Municipality, Eastern Cape Province, South Africa. The plants were then transported to the University of Fort Hare, Department of Biochemistry and Microbiology. They were then separated into leaves and flowers. Subsequently, the plant was authenticated by Dr B Mayekiso at the Department of Botany, University of Fort Hare and the plant was deposited with a voucher specimen UFH2018060 in their herbarium.

### Plant preparation and extraction

Plant preparation and extraction were carried out as described with some modifications. The leaf and flower parts of *L.* *leonurus* were rinsed twice with double-distilled water, air-dried for 2 weeks, thereafter pulverized into powder using an electric blender. Subsequently, 100 g of each part of the plant was weighed and extraction was carried out using solvents of increasing polarity, also referred to as sequential extraction. For extraction of bioactive compounds, extracts were mixed with acetone, methanol and chloroform separately. The mixture was incubated at 25 °C for 24 h maintaining the shaker speed 200 rpm. Each plant extract was filtered using Whatman No. 1 filter paper and then concentrated using IKA rotary evaporator and subsequently dried at 25 °C.

### Phytochemical compounds screening

The bioactive compounds such as total phenolic, flavonoids and alkaloids were determined according to standard procedures (Adedapo et al. 2008; Otang et al. 2012; Yadav and Agarwala 2011).

### Antioxidant assays

#### DPPH scavenging activity

The antiradical activity of plant extracts against DPPH was estimated using the Brand-Williams et al. (1995) method. The method was conducted by preparing different concentrations (0.05—0.25 mg/mL) of each extract and the standard. DPPH (0.02 mM) was prepared in methanol. Five hundred microlitres (500 µL) of each sample solution and 250 µL DPPH (0.02 mM) were mixed. The resultant solution was mixed properly and left in the dark for 30 min. The control was made by mixing ethanol and DPPH. A reference compound was made by mixing ascorbic acid with DPPH. Thereafter, the absorbance of each mixture was read at a wavelength of 517 nm using Ultrospec Visible Plate Reader II.

The scavenging potential was estimated with the following formula:

$$ {\text{DPPH antiradical activity }}\left( \% \right)\, = \,\left( {\frac{{{\text{ABS}}_{{{\text{control}} - {\text{ABS}}_{{{\text{sample}}}} }} }}{{{\text{ABS}}_{{{\text{control}}}} }}} \right) \times 100. $$

${\text{ABS}}_{{{\text{control}}}}$ and ${\text{ABS}}_{{{\text{sample}}}}$ denote the absorbance values of the control as well as the sample material. The 50% inhibitory concentration (${\text{IC}}_{50} )$ values were determined to estimate the concentration of a sample/standard needed to scavenge 50% of the DPPH (0.02 mM) radical. The reference compound used as ascorbic acid.

#### Reducing ability

Ferric reducing the potential of the extracts was estimated using the method of Oyaizu (1986), with some modification. The method was carried out by preparing different concentrations (0.05–0.25 mg/mL) of each extract as well as the standard. A volume of 500 µL of each sample, 1.25 mL of phosphate buffer (0.2 M, pH 6.6) and 1.25 mL of K_3_ [Fe(CN)_6_] (1% w/v) were mixture together. Thereafter, the resultant mixture was incubated at 50 °C for 20 min ${\text{prior to adding}}$ 1.25 mL of 10% (v/v) C_2_HCl_3_O_2_ and centrifuging the mixture at 3000 rpm, 50 °C for a period of 10 min. Thereafter, an upper layer of 1 mL was removed and mixed with 2 mL distilled water, before determining the absorbance at 700 nm using the Ultrospec Visible Plate Reader II. Ascorbic acid was used as standard compound.

#### ABTS·^+^ scavenging activity

ABTS radical scavenging potential of the plant extracts was carried out in accordance with the description of Adedapo et al. (2008). The ABTS·+ was prepared by mixing equal volumes of ABTS (7 mM) and K_2_S_2_O_8_ (2.4 mM) solutions and incubating the mixture in the dark at 25 °C for a period of 12 h. The resultant solution was diluted by mixing with methanol to get a final absorbance value of 0.706 ± 0.001 units at 734 nm. One hundred microliter (100 µL) from various concentrations (0.05–0.25 mg/mL) of each plant extract as well as the standard (BHT) was added to 250 µL of the ABTS·+ radical and left to react in the dark for 7 min. The absorbance was then measured at a wavelength of 734 nm in an Ultrospec Visible Plate Reader II. The ABTS·^+^ scavenging effect of the plant extract was calculated along with the reference compound (BHT) using the following equation:

$$ {\text{ABTS}}^{ \cdot + } {\text{antiradical activity}}\, = \,\frac{{{\text{Abs}}_{{{\text{control}}}} - {\text{ Abs}}_{{{\text{sample}}}} }}{{{\text{Abs}}_{{{\text{control}}}} }} \times 100, $$

where ${\text{Abs}}_{{{\text{control}}}} { }$ and ${\text{Abs}}_{{{\text{sample}}}}$ denote the absorbance of ABTS radical + ethanol as well as the ABTS radical + sample extract or standard butylated hydroxytoluene (BHT).

#### Hydrogen peroxide scavenging activity

Hydrogen peroxide (${\text{H}}_{2} {\text{O}}_{2} )$ antiradical activity of each plant sample was determined using the method reported by Oyedemi et al. (2010). From each stock solution of extract/standard, concentrations of 0.7, 1.4 and 2.1 mg/mL were prepared by further diluting with ethanol. Subsequently, 2 mL of each sample/standard and 0.6 mL of 4 mM ${\text{H}}_{2} {\text{O}}_{2}$ solution prepared in phosphate buffer (0.1 M, pH 7.4) were mixed together. The resultant mixture was subjected to incubation at room temperature for a period of 10 min. The absorbance of the solution was then read at a wavelength of 230 nm using Thermo Spectronic Biomate 3 spectrophotometer. A blank containing phosphate buffer + ${\text{H}}_{2} {\text{O}}_{2}$ without the plant extract while ascorbic acid was used as the reference compound. The capacity of an extract to inhibit the ${\text{H}}_{2} {\text{O}}_{2}$ radical was estimated using the following equation:

$$ {\text{H}}_{2} {\text{O}}_{2} {\text{ antiradical activity}} = { }\frac{{{\text{Abs}}_{{{\text{control}}}} - {\text{ Abs}}_{{{\text{sample}}}} }}{{{\text{Abs}}_{{{\text{control}}}} }} \times { }100. $$

${\text{Abs}}_{{{\text{control}}}}$ and ${\text{Abs}}_{{{\text{sample}}}}$ denote the absorbance of ${\text{H}}_{2} {\text{O}}_{2}$ and ${\text{H}}_{2} {\text{O}}_{2}$ radical + sample extract respectively.

#### Nitric oxide radical scavenging activity

This property of the plant extracts was assessed using Griess ilosvay reaction (Garrat 1964). Griess ilosvay reagent was prepared using 0.1% (w/v) napthyl ethylene diamine dihydrochloride in place of 5% (w/v) 1-napthylamine and subsequently as follows: A total volume of 3 mL was prepared by mixing 2 mL sodium nitroprusside (10 mM), 0.5 mL saline phosphate buffer and 0.5 mL of standard solution or plant extract (0.2–0.8 mg/mL). The reaction mixture was incubated at 25 °C for a period of 150 min. Thereafter, 1 mL from sulfanilic acid reagent (0.33% w/v, in 20% glacial acetic acid) was added to 0.5 mL of the reaction mixture and incubated for 5 min at room temperature for the diazotization reaction to go to completion. Following that, 1 mL napthyl ethylene diamine dihydrochloride was added to the reaction mixture. The resultant reaction mixture was allowed to stand for a period of 30 min at 25 °C. The produced nitrite concentration was assessed by reading absorbance at a wavelength of 546 nm using Ultrospec Visible Plate Reader II. The scavenging effect was estimated using the following formula:

$$ {\text{Nitric oxide radical scavenging activity}}\, = \,\frac{{{\text{Abs}}_{{{\text{control}}}} - {\text{ Abs}}_{{{\text{sample}}}} }}{{{\text{Abs}}_{{{\text{control}}}} }} \times 100, $$

where ${\text{Abs}}_{{{\text{control}}}}$ is the absorbance of the control absorbance of the standard nitrite solution without extracts or reference compound while ${\text{Abs}}_{{{\text{sample}}}}$ is the absorbance of the sample.

### Cytotoxicity assessment

The cytotoxic potential of plant extracts was evaluated against HeLa cells (Cellonex, South Africa) as described by Okaiyeto et al. (2019). The crude extracts were dissolved in dimethyl sulfoxide (DMSO) and it is noteworthy that all the solvent extracts dissolved completely in DMSO and subsequently, the crude extracts were incubated at 37 °C for 48 h at a fixed concentration of 50 µg/mL in 96-well plates containing HeLa cells. Emetine was used as positive control drug and the cells that survived plant extracts and cells exposure were quantified by incubation with resazurinand measuring its conversion to resorufin by fluorescence (Exc560/Em590). Percentage viability of treated cells was calculated relative to fluorescence readings obtained with untreated control cells.

### Gas chromatography-mass spectrometry (GCMS) analysis

Fifty microliter (50 µL) of plant extract samples was diluted with 4.95 mL of ethanol and injected into a GCMS system consisting of Agilent 7890B GC System coupled with 5977A mass spectra data (MSD. An HP-5 fused silica capillary column (30 m × 0.320 mm i.d. and 0.250 μm film thickness) was used. The injector was set at 250 °C, 48.745 kPa and run at pulse splitless mode. The injection volume of the sample was 1 μL. The carrier gas, helium (99.999% purity) was run at average velocity: 36.262 cm/s. Oven Temperature Programming was started from 40 °C (held for 1 min), ramped to 240 °C at 3 °C/min. The total runtime was 67.667 min. The GCMS data was analysed with 'MassHunter' software and the (possible) compounds obtained were identified by comparing their retention time with those of authentic compounds and the ones from spectral data.

### Statistical analysis

Data generated from phytochemical and antioxidant studies were expressed as mean values ± standard deviation. For the cytotoxicity assays, IC_50_ values were derived by non-linear regression analysis using GraphPad Prism.

## Results and discussion

Medicinal plants have remarkably become the centre of attention in scientific research mainly due to the role they play in the livelihoods of indigenous people (Dar et al. 2017). They have been extensively utilised in traditional practice settings to treat a variety of diseases including cancer, diabetes, hepatitis, tuberculosis and neurodegenerative disorders (Sofowora et al. 2013). Another striking aspect of medicinal plants is the possession of a wide variety of bioactive compounds with fewer side effects compared to synthetic antioxidants (Sasidharan et al. 2011). As a result thereof, the present study investigated the properties of *L.* *leonurus* since it is one of the extensively used medicinal plants against a number of health conditions in South Africa.

### Total phenolic, flavonoids and alkaloids determination

**Fig. 1:**
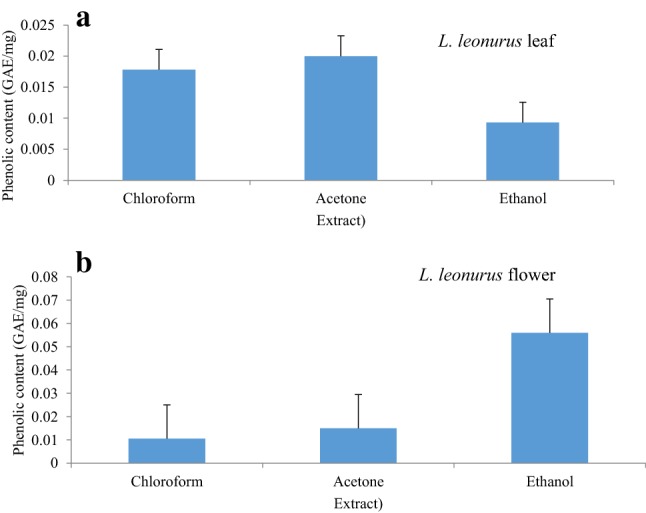
Phenolic content of *L.* *leonurus* leaf (**a**) and flower (**b**)

In the present study, high phenolic content was observed in acetone and chloroform leaf extracts of *L.* *leonurus* with close values of 0.02 and 0.0178 mg/g (GAE), respectively (Fig. 1a). These results corroborate the report of Jimoh et al. (2010) which revealed high phenolic and flavonoid content in acetone, methanol and aqueous extracts of *L.* *leonurus*. Leaf ethanolic extract displayed the lowest phenolic content of 0.0093 mg/g (GAE). With the flower part of *L.* *leonurus* plant, the ethanolic extract showed high phenolic content (Fig. 1b) of 0.056 mg/g (GAE) as compared to acetone (0.015 mg GAE) and chloroform (0.106 mg/g GAE) extracts. With these findings, it can be deduced that ethanol was the most effective solvent to extract phenolic acids. Further, it has been reported that ethanolic extract of Ivorian plants displayed a higher amount of phenolics compared to acetone, aqueous, and methanolic extracts (Koffi et al. 2010).

**Fig. 2:**
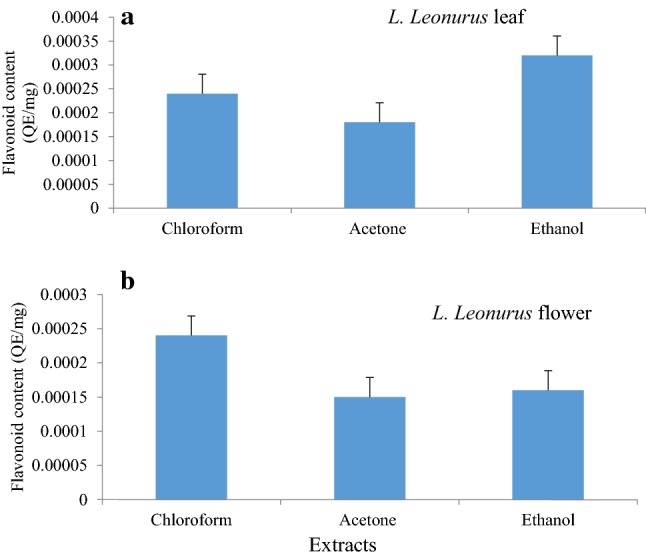
Flavonoid content of various *L.* *leonurus* leaf (**a**) and flower (**b**) extracts in different solvents. The concentration of flavonoid content from each extract was calculated from the standard curve as milligrams equivalents of quercetin per gram

High flavonoid content was observed in the ethanolic extract of the leaf followed by chloroform and acetone extracts with values of 0.00032, 0.00024, 0.00018 mg/g quercetin equivalent (QE), respectively (Fig. 2a). With flavonoid content, the chloroform flower extract had the highest flavonoid content followed by the ethanolic extract and acetone extract with values of 0.00024, 0.00016, 0.00015 mg/g QE, respectively (Fig. 2b). However, the ethanolic extract of the flower revealed higher phenolic content compared to leaf extracts (Fig. 1a, b). On the other hand, flavonoid content was more abundant in the leaf ethanolic extract than the flower extract (Fig. 2a, b). From these observations, it can be deduced that ethanol is the most effective solvent to extract polar compounds from plant extracts.

In this present study, the presence of alkaloids from all extracts was confirmed by the appearance of orange to red turbid suspension when reacted with Mayer’s and Wagner’s reagents. The appearance of this positive reaction in all extracts served as strong indication and confirmation of *L.* *leonurus* as a potential source of alkaloids. This finding corroborates the study conducted by Bienvenu et al. (2002) which confirmed the presence of alkaloids by the formation of turbidity. The principle underlying this method is the ability of alkaloid compounds, possibly present in the plant extracts, to couple their nitrogen atom with the heavy metal ions present in Mayer’s and Wagner’s reagent to form ion pairs that result in an insoluble yellow–red precipitate (Coe and Anderson 1996).

The presence of phenolic, flavonoid and alkaloid compounds in these plant extracts is an indication that the plant studied might have antioxidant potentials to scavenge free radicals. This view is supported by the fact that antioxidant properties observed in vegetables, fruits and herbs are largely due to the presence of compounds such as phenolic acids and flavonoids (Shi et al. 2003; Lee et al. 2017). These compounds are also reported to have crucial leading roles in plant development. Phenolics are reported to be involved in plant growth, resistance, as well as plant defence against microbial infections that are intrinsically related to oxidative stress (Grassmann et al. 2002). Flavonoids also exert a crucial role in the plant defence system with their rapid scavenging ability during pathogen invasion (Panche et al. 2016). This action is intended to kill bacterial, metastatic, or virus-infected intruders (Kujumgiev et al. 1999; Limasset et al. 1999; Havsteen 2002). Alkaloids are mainly produced from precursors such as amino acids or their immediate derivatives during environmental stress, herbivore attack and pathogen invasion or in times of plant growth and development (Aniszewski 2006). *L.* *leonurus* has proven to be a potential source of phenolics, alkaloids and flavonoids. Other studies have also confirmed the phytochemical properties of the aqueous extract of *L.* *leonurus* are largely due to the presence of flavonoids and alkaloids (Laonigro et al. 1979; Bienvenu et al. 2002; Mazimba 2015). Another study conducted by El-Ansari et al. (2009) had successfully isolated flavonoids from the flowering aerial parts of *L.* *leonurus* using aqueous, alcoholic and chloroform extracts.

### DPPH scavenging activity

**Fig. 3:**
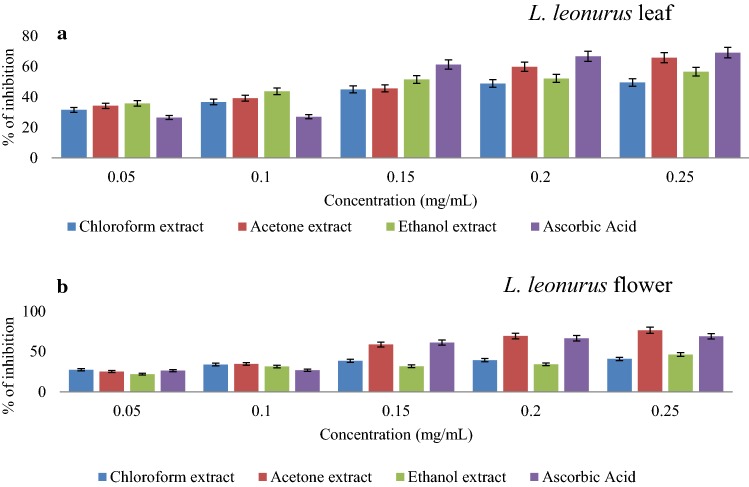
DPPH radical scavenging capability of various *L.* *leonurus* leaf (**a**) and flower (**b**) extracts with increasing concentration in different solvents

DPPH scavenging activities of the extracts are presented in Fig. 3a, b. The results revealed that all extracts, as well as the standard, followed a similar trend where an increase in concentration resulted in an increase in scavenging activity. Acetone leaf extract displayed the highest scavenging activity (65.8%) at 0.25 mg/mL (Fig. 3a). More evidence that supports the DPPH scavenging activity of this extract is the fact that its inhibition is very close to that of ascorbic acid, which is 69.12% at 0.25 mg/mL. A similar finding was reported in another study carried out by Jimoh et al. (2010) where acetone extract of *L.* *leonurus* leaf had the highest DPPH scavenging activity as compared to both methanol and aqueous extracts. It was further noted that ethanolic extract of the leaf exhibited the second highest scavenging activity (56.62%) at 0.25 mg/mL while chloroform leaf extract recorded the least DPPH scavenging potential at approximately 50% at 0.25 mg/mL. The acetone extract had the lowest ${\text{IC}}_{50}$ value of 3.13 mg/mL as compared to ethanolic (4.6 mg/mL) and chloroform (3.48 mg/mL) extracts. Furthermore, there was a positive correlation observed between phenolic content and DPPH radical scavenging activity of acetone leaf extracts. This is where leaf acetone extract displayed the highest phenolic content as well as DPPH scavenging activity than other extracts. Such findings seem to support the claim that phenolic compounds in acetone extract largely contribute to the observed DPPH antiradical activity of *L.* *leonurus* leaf.

Flower extracts exhibited higher scavenging activity compared to leaf extracts with percentage scavenging activity of 76.51% recorded for acetone extract at 0.25 mg/mL, a scavenging activity higher when compared to the standard ascorbic acid (Fig. 3b). A similar pattern of scavenging activity observed with leaf extracts was also noticed with flower extracts whereby the ethanolic extract showed the second-highest scavenging activity at 46.47%, with the lowest (40.96%) obtained for chloroform extract, all at 0.25 mg/mL (Fig. 3b). The DPPH scavenging potential of bioactive compounds in an extract is related to their hydrogen donating ability which confers the antioxidant power (Yu et al. 2002; Jimoh et al. 2010). These results further support the claim that acetone is one of the most effective solvents to extract bioactive compounds from plants (Jimoh et al. 2010). Most published studies have been carried out on leaf, roots and stems of *L.* *leonurus* plants with less attention being paid to the flower section, thus, this study reveals the possibility that the flower might also be a rich source of antioxidants and bioactive compounds.

### Reducing ability

**Fig. 4:**
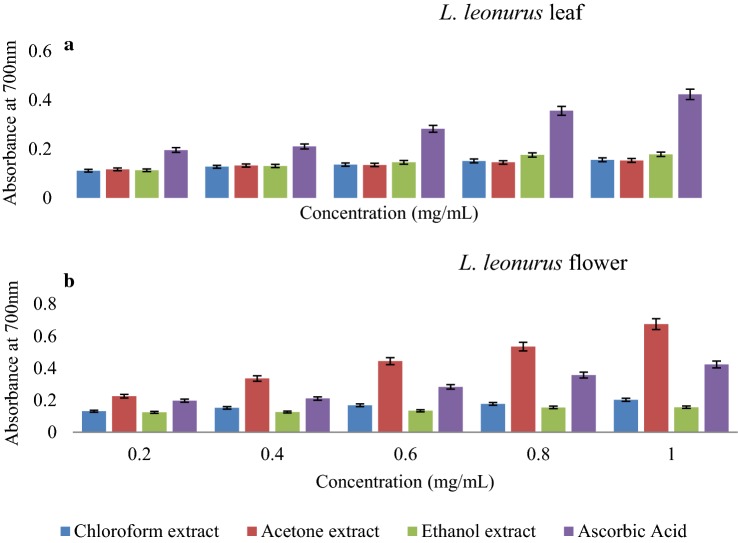
Reducing abilities of *L.* *leonurus* leaf (**a**) and flower (**b**) extracts in solvents of increasing polarity compared with ascorbic acid as a standard compound at various concentrations

It has been previously documented that an effective reducing power of an extract is a reflection of the antioxidant property a plant may possess (Do et al. 2014). The ferric reducing assay is one of the mostly used methods developed to test the presence of electron donors in plant extracts which are considered to be important in human health (Gülçin 2015). Figure 4a, shows that from 0.2 to 0.4 mg/mL, leaf acetone extracts had a slightly higher ferric reducing power, followed by ethanolic and chloroform extracts. At different concentrations (0.6–1 mg/mL), ethanolic extract showed higher reducing ability compared to other extracts. Furthermore, Fig. 4b depicts the reducing ability of extracts from the *L.* *linorus* flower. Acetone extract exhibited the highest reducing power at the concentrations tested, followed by chloroform extract. Ethanolic extracts showed the lowest reducing ability. From the extracts of both studied plant parts, acetone and ethanolic extracts displayed better reducing potential and these could be linked to their high phenolic and flavonoid contents. The flower plant part demonstrated more effective ferric reducing ability than the leaf part. The findings of this study revealed the ability of these plant extracts to reduce ${\text{Fe}}^{3 + }$ to ${\text{Fe}}^{2 + }$, which is an indication of their potential role as antioxidant agents which needs more attention in the research field of medicine. Although iron is required for normal physiological function in the brain, however, dysregulation in iron homeostasis may lead to neurotoxicity via induction of overproduction of OH radicals and initiation of lipid peroxidation as well as deprivation of the brain tissues from other essential metals and initiation of protein aggregation within the neurons (Singh et al. 2014; Olasehinde et al. 2019). Therefore, metal chelation therapy has been recommended as one of the therapeutic approaches for the management of neurodegenerative disease.

### ABTS radical scavenging activity

**Fig. 5:**
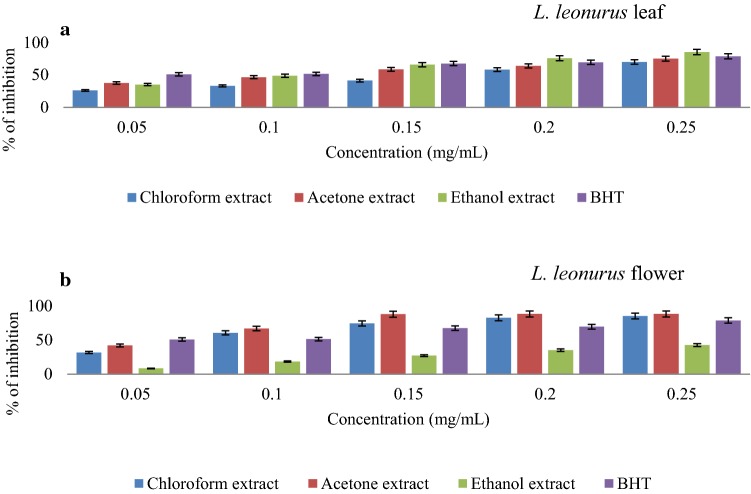
ABTS radical scavenging activity of *L.* *leonurus* leaf (**a**) and flower (**b**) extracts with increasing concentrations, compared with standard BHT

The ability of an extract to scavenge protonated radicals is an important property that relates to its antioxidant ability (Wolf et al. 2016). ABTS·^+^ is a protonated radical that was used to assess the antioxidant property of *L.* *leonurus*. All leaf extracts studied exhibited a concentration-dependant ABTS·^+^ radical scavenging activity (Fig. 5a). The ethanolic extract displayed the highest inhibition (over the concentration range 0.05–0.25 mg/mL) compared to other extracts and the BHT standard, an indication of the possible presence of potent compounds with high antioxidant activity.

Figure 5b depicts a similar antioxidant capability of *L.* *leonurus* flower extracts that was observed in DPPH radical scavenging activity. This indicates that flower acetone extract exhibited high ABTS·^+^ scavenging activity than the other extracts, but slightly below that of the positive control (BHT). The ${\text{IC}}_{50}$ (0.823 mg/mL) of flower acetone extract is the lowest amongst the sample extracts tested, as well as the standard. *L.* *leonurus* chloroform flower extract exhibited the second highest ABTS·^+^ radical activity, after acetone. It displayed its highest inhibition of 85% at 0.25 mg/mL. The ethanolic extract exhibited a similar trend of concentration dependent increase albeit at a lower inhibition rate compared to other extracts (Fig. 5b). The ${\text{IC}}_{50}$ value of the ethanolic extract was found to be 5.764 mg/mL, an indication of its less effectiveness.

### Hydrogen peroxide radical scavenging activity

**Fig. 6:**
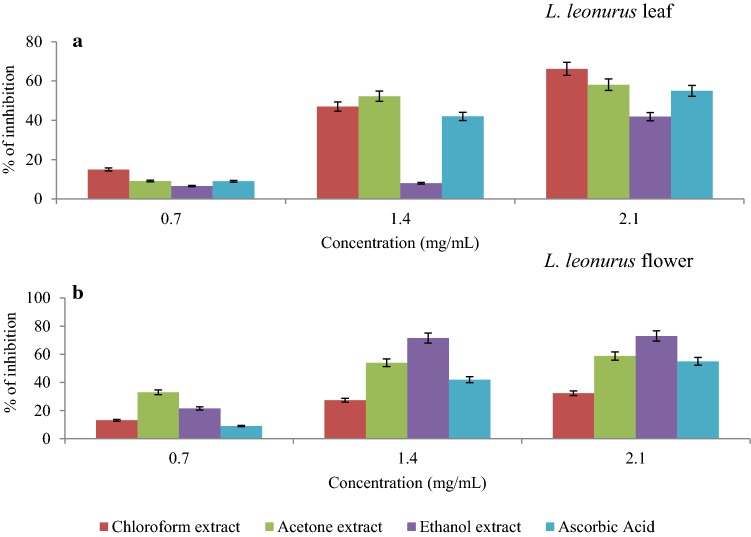
Hydrogen peroxide scavenging activity of various *L.* *leonurus* leaf (**a**) and flower (**b**) extracts in solvents of increasing polarity

Although hydrogen peroxide is regarded as a non-radical compound, it has been reported as a biological source of a very reactive radical called hydroxyl ion (Mokudai et al. 2012). This means supra physiological amounts of hydrogen peroxide greatly contribute to oxidative stress (Sies 2017). In essence, if a plant derived antioxidant expresses scavenging activity against hydrogen peroxide, less hydroxyl ions would be generated during oxidative stress. *L.* *leonurus* extracts in this study demonstrated effective scavenging activity against hydrogen peroxide, in a dose-dependent manner. Acetone and chloroform leaf extracts of *L.* *leonurus* exhibited high inhibition scavenging activity against hydrogen peroxide compared to ethanolic extract as well as the standard ascorbic acid (Fig. 6a). The scavenging activities of acetone and chloroform leaf extracts of *L.* *leonurus* recorded at 2.1 mg/mL were 58.1% and 66.2%, respectively, whereas, ethanolic extract and ascorbic acid, were observed to be 35.5% and 55% (Fig. 6a). The lower ${\text{IC}}_{50}$ (mg/mL) values are further evidence in support of the inhibitory effect of chloroform extract against hydrogen peroxide compared to other extracts. The flower extracts of *L.* *leonurus* also showed inhibition towards hydrogen peroxide. The difference from the leaf part is that the ethanol extract was in comparison more effective than the acetone and chloroform extracts. From Fig. 6b, the highest inhibition of 73% was observed for ethanolic extract at 2.1 mg/mL, while acetone and chloroform extracts were 58.8% and 32.4%, respectively. The study of Valko et al. (2007) highlighted that OH is a highly reactive radical that initiate lipid peroxidation via the removal of an electron from poly-unsaturated fatty acids, which leads to the formation of peroxyl radicals. Furthermore, Uttara et al. (2009) revealed that excessive production of free radicals has been associated with oxidative damage that consequently leads to oxidative stress in the neurons. There is still a lack of information about the hydrogen peroxide scavenging activity of *L.* *leonurus* plants; hence, the present study contributes towards knowledge on their antioxidant abilities.

### Nitric oxide scavenging activity

**Fig. 7:**
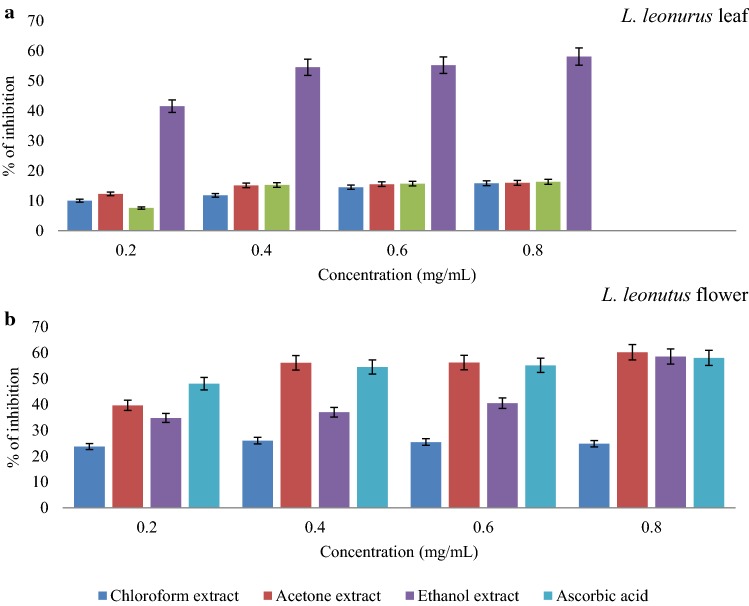
Nitric oxide scavenging ability of *L.* *leonurus* leaf (**a**) and flower (**b**) extracts in different solvents, compared with ascorbic acid

Reactive nitrogen species have gained enormous attention in the research field of medicine due to the role they play in normal physiological processes as well as their contribution towards alleviating oxidative stress (Heras et al. 2001). Additionally, the reaction of superoxide and nitric oxide yields peroxynitrite, which is a very strong oxidant that can indiscriminately react with any biomolecule, also inhibiting important enzymes that will affect the integrity of mitochondrion (Turrens 2003). With regards to *L.* *leonurus* leaf extracts, it was discovered that all extracts were able to scavenge nitric oxide but with a very limited ability. As illustrated in Fig. 7a, none of these extracts was able to effectively scavenge nitric oxide above 50%. Within that range, the acetone extract had the highest scavenging activity against the nitric oxide radical. Moreover, the ${\text{IC}}_{50}$ (mg/mL) values of chloroform, acetone and ethanolic extracts were 20.89, 33.19 and 16.09 mg/mL respectively. The flower extracts of *L.* *leonorus* (Fig. 7b) showed better results than the leaf extracts. All flower extracts were able to scavenge the nitric oxide radical at the concentration tested.

The acetone extract of the flower had the highest scavenging activity which was similar to that of the standard (ascorbic acid). The highest inhibition rate of the acetone flower extract was 60% at 0.8 mg/mL while that of ascorbic acid was 58% (Fig. 7b). This means that the flower acetone extract had a comparable nitric oxide radical scavenging activity to that of the reference compound. Following the acetone extract, the flower ethanolic extract showed the second maximum with an inhibition rate of 58.6% at 0.2 mg/mL. The flower chloroform extract was unable to inhibit above 25% and this result means that the bioactive compounds extracted with this solution were not effective inhibitors compared to other extracts. The results further support the idea that the flower of *L.* *leonurus* possibly contains phytochemicals with the ability to scavenge radicals implicated in oxidative stress more than the leaf part. Accordingly, *L.* *leonorus* was shown to possess antioxidant effect against radical oxidants, as such, *L.* *leonurus* flower has emerged as the major likely source of antioxidants.

### GCMS analysis

GCMS analyses of plant extracts showed the presence of a wide range of compounds associated with antioxidant and acetylcholinesterase (AChE) inhibitory properties. Compounds that were identified (Tables 1, 2) to be present in large amounts include; 3-methyl-4-(3,7,7-trimethyl-2-oxabicyclo[3.2.0]hept-3-en-1-yl)-but-3-en-2-one (36.8%), carbofuran (14.56%) and phenol, 2-methoxyl-4-(1-propenyl)-trans-isoeugenol (14.56%) from acetone, chloroform and ethanolic extracts, respectively. Other compounds that were found in significant amounts included 2, 4-di-*tert*-butylphenol, *n*-nonadecanol, phytol, 9,12-octadenoic acid, 1-octadecene, undulatine, eugenol. In the flower extract, the major compounds that were identified included; 1-octadecene (14.16%), 3-eicosene (12.82%), 2-tetradecene (11.31%), 1-nonadecene (10.75%), 1-hexacosene (10.75%), undulatine (8.02%) and cyclotetracosene (9.74%). GC–MS analysis of chloroform flower extract also revealed the presence of undulatine, a compound known to possess neuroprotective properties (van Rijn et al. 2010; Cahlíková et al. 2013).

**Table 1:** GC–MS analysis of *L.* *leonurus* leaf solvent extracts

Component^a^	Chemical formula^b^	RT^c^	Composition^d^ (%)			QA^e^
			Acetone extract	Ethanol extract	Chloroform extract	
2,4-Di-tert-butylphenol	C_14_H_22_O	34.111	–	3.76	1.16	97
*n*-Nonadecanol	C_19_H_40_O	50.915	–	–	5.43	94
Phytol	C_20_H_40_O	54.524	3.97	5.16	8.30	99
9.12 Octadenoic acid (z,z)	C_18_H_32_O_2_	55.216	0.45	–	4.21	97
1H-indene-1,3(2H)-dione,2-[2,3,6,7-tetrahydro-1H,5H-benzo[i]quinolizin-9-yl)methylene-	Un	59.904	5.12	–	–	68
Undulatine	C_18_H_21_NO_5_	61.034	3.76	–	–	99
1-Octadecene	CH_3_(CH_2_)_15_CH=CH_2_	62.425	–	6.51	–	98
4-Methoxy-2-allyphenol	C_10_H_12_O_2_	63.187	–	–	8.04	59
Eugenol	C_10_H_12_O_2_	63.433	–	–	3.1	64
Phenol,2-methoxyl-4-(1-propenyl)-trans-isoeugenol	C_10_H_12_O_2_	65.361	–	–	14.56	64
Carbofuran	C_12_H_15_NO_3_	65.361	–	14.56	0.77	64
3-Methyl-4-(3,7,7-trimethyl-2-oxabicyclo[3.2.0]hept-3-en-1-yl)-but-3-en-2-one	C_13_H_18_O_2_	65.491	36.8	–	–	53

*Ui* unidentified, *Un *unknown

^a^Component/compound

^b^Chemical formula

^c^Retention time

^d^Composition in percentage (%)

^e^Quality assurance of GC/MS library

**Table 2:** GC–MS analysis of *L.* *leonurus* flower solvent extracts

Componenta	Chemical formulab	RTc	Compositiond (%)			QAe
			Acetone extract	Ethanol extract	Chloroform extract	
2,6-Dimethyl-3-ethyl-pyridine	C_9_H_13_N	16.002	4.84	–	–	84
2-Tetradecene	C_14_H_28_	29.302	–	–	6.01	98
2.5-bis(1,1-dimethylethyl) phenol	Un	34.281	–	–	11.31	95
2-Tetradecene, Cetene	C_14_H_28_	37.123	–	–	11.31	98
1-Octadecene	CH_3_(CH_2_)_15_CH=CH_2_	44.427	–	–	14.16	96
3- Eicosene	C_20_H_40_	50.970	–	–	12.82	96
Phytol	C_20_H_40_O	54.526	0.24	2.17	–	99
1-Nonadecene	C_19_H_38_	56.967	–	–	10.75	99
1-Hexacosene	C_26_H_52_	57.929	–	10.75	–	95
Undulatine	C_18_H_21_NO_5_	61.040	–	–	8.02	99
Cyclotetracosane	C_24_H_48_	62.49	–	–	9.74	98

*Ui* unidentified, *Un* unknown

^a^Component/compound

^b^Chemical formula

^c^Retention time

^d^Composition in percentage (%)

^e^Quality assurance of GC/MS library

According to literature, the compound, 3-methyl-4-(3,7,7-trimethyl-2-oxabicyclo[3.2.0]hept-3-en-1-yl)-but-3-en-2-one is a terpernoid that is suspected to exhibit antioxidant and AChE inhibitory properties (Ahmad et al. 2016). Carbofuran is commonly used in agriculture as an insecticide and is known to possess the ability to inhibit AChE (Gupta 1994; Tennakoon et al. 2009). On the other hand, literature is still scanty about the biological function of phenol, 2-methoxyl-4-(1-propenyl)-trans-isoeugenol. Some of these compounds have been documented to show excellent antioxidant and antiacetylcholinesterase properties. In a study by Yoon et al. (2006) 2,4-di-*tert*-butylphenol was found to possess good antioxidant activity against low-density lipoprotein (LDL) oxidation (Yoon et al. 2006). Phytol is another compound reported to possess antioxidant abilities. An in vitro study carried out by Santos et al. (2013) to assess the antioxidant activity of phytol showed that this compound was able to scavenge hydroxyl radical and nitric oxide. Undulatine, alongside with other amaryllidaceae alkaloids isolated from *Chlidanthus* *fragrans* herb, has been reported to inhibit acetylcholinesterase activity (Cahlíková et al. 2013). Furthermore, the anticancer and antioxidant activity of 1-octadecene has been reported (Renukadevi et al. 2011). Eicosene and 2-tetradecene, are compounds that are suspected to contribute to the anticancer, antimicrobial and antioxidant ability of plant extracts (Manoj et al. 2012; Tiloke et al. 2018).

Moreover, the presence of these compounds explains the antioxidant abilities of *L.* *leonurus* leaf and flower extracts observed in this study. Antioxidant capacity of natural bioactive compounds has generated interest in medicine and research. The anti-inflammatory compounds discovered in this study are further proof of the beneficial properties *L.* *leonurus* possesses. Additionally, the discovery of such compounds as well as their reported properties increase the understanding as well as providing an insight into why indigenous people value this plant as it has numerous applications related to health. The presence of AChE inhibitors in the composition of *L.* *leonurus* flower is a significant discovery that needs close attention as this evidence highlights the significance of *L.* *leonurus* in the treatment of neurodegenerative diseases.

### Cytotoxicity assessment

**Fig. 8:**
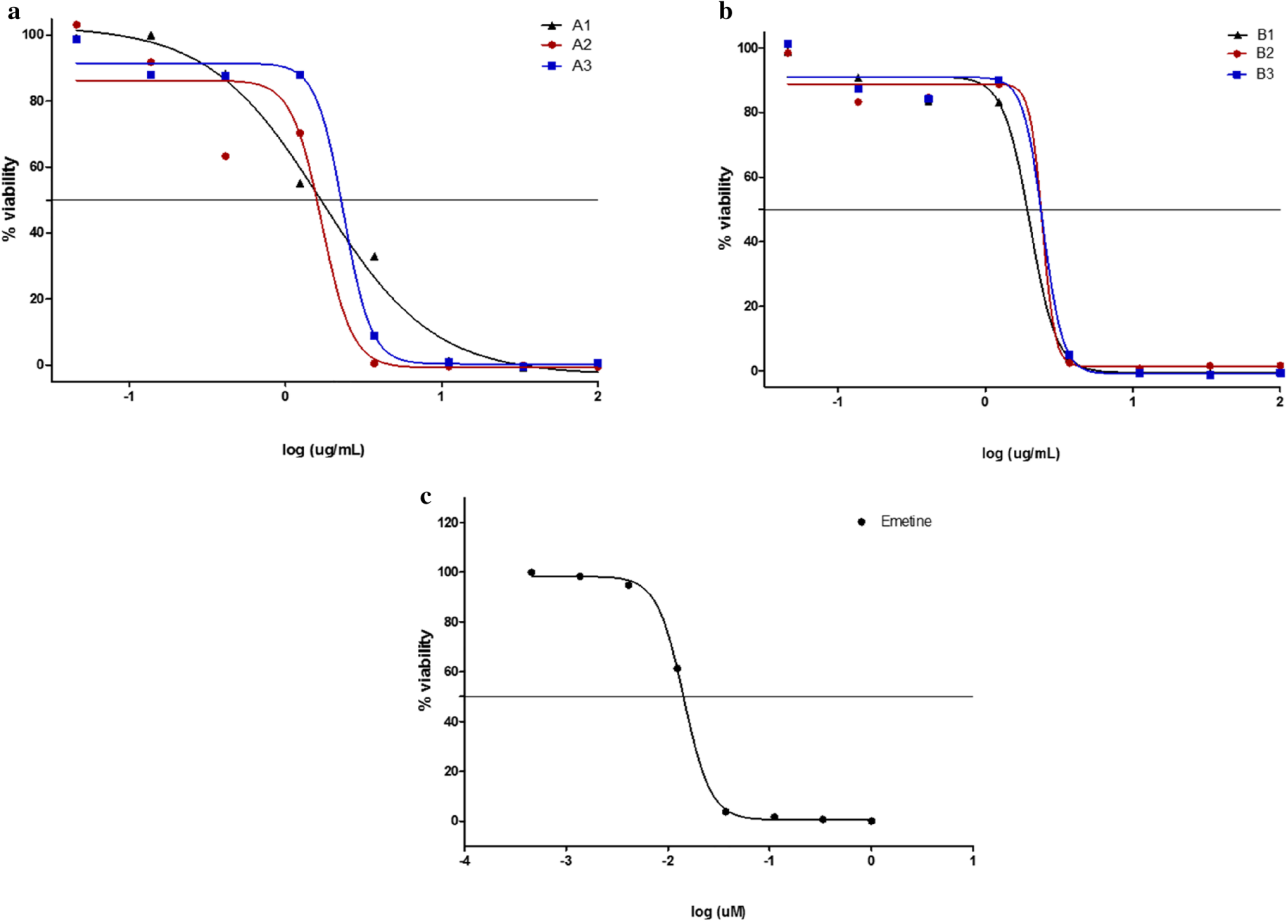
Dose–response graphs of the effect of leaf (**a**) and flower (**b**) extracts on HeLa cell viability. Each data point represents the mean of technical duplicates. A1: *L.* *leonurus* leaf chloroform; A2: *L.* *leonurus* leaf acetone; A3: *L.* *leonurus* leaf ethanolic extract. B1-3 are the corresponding extracts from the flowers of *L.* *leonurus*. Emetine was used as a positive control (**c**)

Cytotoxicity assessment is a very critical aspect of drug discovery from plant origin. This is mainly due to the fact that it elucidates information about the safety of drugs, as well as precautionary measures that should be considered. As illustrated in Fig. 8a, b, as the concentration of extracts increases, cell viability declines. At a low concentration of about 0.04 µg/mL, acetone, ethanol and chloroform extracts from the leaf of *L.* *leonorus* showed negligible effect on cell viability with % viability ranging between 85 and 100%. However, an increase in extract concentration led to a decline in cell viability for chloroform extract (A1) compared to the other two extracts (A2 and A3) which did not affect cell viability until a concentration of about 0.41 µg/mL was reached after which a sharp decline in % viability was observed. It is further observed that at a concentration of 3.7 µg/mL, A1 produced a viable cell population of 32.97%. Extract concentrations above 1.23 µg/mL seem to be lethal to HeLa cells with a complete loss of viability at concentrations above that. The ${\text{IC}}_{50}$ values of these extracts were calculated to be 1.7 µg/mL, 1.6 µg/mL and 2.4 µg/mL for A1, A2 and A3, respectively. Comparing *L.* *leonurus* extracts with emetine (Fig. 8c), a compound known to induce cell apoptosis, it can be deduced that *L.* *leonurus* extracts were less toxic than emetine. The acute cytotoxicity of emetine was observed at 0.01 µg/mL while those of the leaf extracts started at 10.9 µg/mL. A previous study carried out by Kee et al. (2008) on cytotoxicity effect of *L.* *leonurus* tannin-free organic and aqueous extracts noted that compounds from these extracts had high cytotoxicity levels against HL-60 and K562 cell lines. Moreover, one of the compounds (1-decanol, 2-hexyl) identified in ethanolic leaf extract of *L.* *leonurus* is reported to be toxic against the skin. Therefore, the presented findings support the possibility of *L.* *leonurus* compounds being toxic towards mammalian cells.

As for flower extracts, it was also observed that the extract concentration is inversely related to cell viability. Figure 8b shows that in all extracts from 0.04 to 1.23 µg/mL, cells maintained their viability at levels between 100 and 80%. Above this concentration range, a sharp drop in cell viability was observed culminating in total cell death from concentrations just above 3.7 µg/mL. The calculated ${\text{IC}}_{50}$ values of flower extracts were 2.0 µg/mL, 2.4 µg/mL and 2.5 µg/mL for B1 (*L.* *leonurus* flower chloroform), B2 (*L.* *leonurus* flower acetone) and B3 (*L.* *leonurus* flower ethanol respectively). Compared to standard emetine, *L.* *leonurus* flower extracts seem to be less toxic than leaf extracts. The dehydroabietic acid identified in flower ethanolic extract might be one of the compounds contributing to the observed cytotoxicity. A report by Oikari et al. (1983), who carried out a study on the toxicological effects of this compound on fish, revealed that dehydroabietic acid is poisonous against these aquatic animals. Aiyegoro and Okoh (2010) reported that alkaloids contain poisonous properties and, therefore, their presence in plant extracts would increase the cytotoxicity levels of the plant.

## Conclusion

In the present study, the phytochemical compounds, antioxidant and cytotoxicity properties of the leaf and flower parts of *L.* *leonurus* were investigated. The antioxidant activity possessed by the plant extract coupled with the key phytochemical compounds identified by GC–MS demand for further research to elucidate the neuroprotective potential of the plant extracts, which is an on-going study in our research laboratory.
